# Differential Expression of the Demosponge (*Suberites domuncula*) Carotenoid Oxygenases in Response to Light: Protection Mechanism Against the Self-Produced Toxic Protein (Suberitine)

**DOI:** 10.3390/md10010177

**Published:** 2012-01-18

**Authors:** Werner E. G. Müller, Xiaohong Wang, Michael Binder, Johannes von Lintig, Matthias Wiens, Heinz C. Schröder

**Affiliations:** 1 ERC Advanced Grant Research Group at the Institute for Physiological Chemistry, University Medical Center of the Johannes Gutenberg University Mainz, Duesbergweg 6, Mainz D-55128, Germany; Email: wxh0408@hotmail.com (X.W.); korzhev@uni-mainz.de (M.B.); wiens@uni-mainz.de (M.W.); hschroed@uni-mainz.de (H.C.S.); 2 National Research Center for Geoanalysis, 26 Baiwanzhuang Dajie, Beijing 100037, China; 3 Department of Pharmacology, Case Western Reserve University, 10900 Euclid Ave., Cleveland, OH 44160, USA; Email: Johannes.vonlintig@case.edu

**Keywords:** Suberitine, β-carotene, retinal, β-carotene dioxygenase, sponges, *Suberites domuncula*

## Abstract

The demosponge *Suberites domuncula* has been described to contain high levels of a proteinaceous toxin, Suberitine, that displays haemolytic activityIn the present study this 7–8 kDa polypeptide has been isolated and was shown to exhibit also cytotoxic effects on cells of the same species. Addition of retinal, a recently identified metabolite of β-carotene that is abundantly present in *S. domuncula* was found to reduce both the haemolytic and the cell toxic activity of Suberitine at a molar ratio of 1:1. Spectroscopic analyses revealed that the interaction between β-carotene and Suberitine can be ascribed to a reversible energy transfer reaction. The enzyme that synthesises retinal in the sponge system is the β,β-carotene-15,15′-dioxygenase [carotene dioxygenase]. In order to clarify if this enzyme is the only β-carotene-metabolizing enzyme a further oxygenase had been identified and cloned, the (related) carotenoid oxygenase. In contrast to the dioxygenase, the carotenoid oxygenase could not degrade β-carotene or lycopene in *Escherichia coli* strains that produced these two carotenoids; therefore it had been termed related-carotenoid oxygenase. Exposure of primmorphs to light of different wavelengths from the visible spectrum resulted after 3 days in a strong upregulation of the dioxygenase in those 3D-cell aggregates that had been incubated with β-carotene. The strongest effect is seen with blue light at a maximum around 490 nm. It is concluded that the toxin Suberitine is non-covalently modified by retinal, the cleavage product from β-carotene via the enzyme carotene dioxygenase, a light inducible oxygenase. Hence, this study highlights that in *S. domuncula* the bioactive metabolite, retinal, has the property to detoxify its homologous toxin.

## 1. Introduction

Since the discovery of the first clinically relevant secondary metabolite isolated from the sponge *Cryptotethya crypta* (phylum Porifera) [1], 1-β-D-arabinofuranosylthymine [ara-T], a cornucopia of unique chemical compounds has been identified. They all have the potential for industrial development as pharmaceuticals, cosmetics, nutritional supplements, molecular probes, enzymes, fine chemicals, and agrichemicals [2,3,4]. From ara-T two derivatives have been chemically synthesized that proved to be potent anti-leukemic (1-β-D-arabinofuranosylcytosine [ara-C] [5]) or anti-viral (9-β-D-arabinofuranosyladenine [ara-A] [6]) drugs. In contrast to the secondary metabolites, proteinaceous bioactive substances were given less attention despite their presumed higher biological and biotechnological importance [7]. This changed gradually with the first cloning of such a bioactive polypeptide, the hemagglutinin from *Geodia cydonium* [8]. Subsequently, further marine bioactive proteins were identified [9] with the ASABF-type antimicrobial peptide from *Suberites domuncula* being the most recent one [10]. The proteinaceous compounds have, if their genes are known, the advantage over the secondary metabolites that they can be modified straightforwardly by molecular biological techniques [11]. 

Alike the secondary metabolites also the bioactive proteins are used by sponges as defense molecules against attacking pro- and eukaryotic organisms [12]. Until their use for defense, the secondary metabolites remain either compartmented in cellular vesicles, as e.g., avarol [13], or are stored as pro-toxins, as e.g., aeroplysinin [14], and by that remain functionally inactive for the sponge producer. In the bioactive peptides, however, the molecules undergo activation during post-translational modification and extracellular transport [10]. In the present study a novel strategy has been detected in sponges by which a toxic peptide remains functionally inactive for the host producer, by the interaction of the peptide with a low molecular bioactive metabolite. Following our earlier finding that the demosponge *S. domuncula* is rich in retinoids [15], we elucidated recently the basic pathway of the retinoids in this species [16]. The retinoid β-carotene is produced by bacteria that are symbiotically associated with this sponge. This precursor molecule is enzymatically processed by cleavage through the β,β-carotene-15,15′-dioxygenase (carotene dioxygenase) to retinal, followed by oxidation via the retinal dehydrogenase to retinoic acid. The end-product retinoic acid functions as morphogen in the sponge via interaction with the ligand-activated transcription factor, the retinoid X receptor [17]. In the present study we give evidence that retinal interacts with the toxic peptide in *S. domuncula*, with Suberitine. 

The biological active Suberitine has been discovered in *S. domuncula* already in 1906 [18] and was then isolated and purified by Cariello and Zanetti [19]. It has been proposed that this peptide acts as a defense molecule against attacking invaders [20]. The peptide with a size of about 7 kDa is composed of four subunits [19], is neurotoxic and displays hemolytic activity [21,22]. The biological activity of Suberitine is abolished after modification of the cysteinyl and tryptophyl residues within the molecule [23]. Importantly, these authors described later a protein from the same sponge, the blue carotenoprotein, with a similar size like the native Suberitine that has the capacity to bind to carotenoids [24]. In the present study we describe that Suberitine in *S. domuncula* is inactivated after binding to retinal. In order to elucidate the tuned activation/inactivation pathway of Suberitine we studied if the carotenoids themselves control the expression of the gene(s) involved in the formation of retinal. Consequently we screened for additional genes that might encode enzymes that cleave carotenoids [25]. We identified two further (potential) carotenoid oxygenases that, however, after expression, did not cleave β-carotene. Therefore, these enzymes had been termed related-carotenoid oxygenases. The data presented here indicate that low molecular bioactive compound(s) that are produced by associated microorganisms and processed by sponge host-encoded enzymes control the biological activity of bioactive peptides in sponges.

## 2. Results

### 2.1. Suberitine: Purification

**Figure 1:**
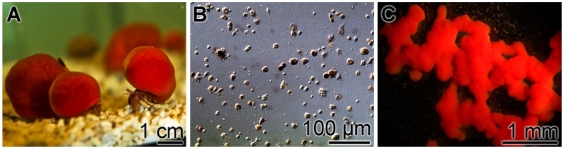
The sponge *S. domuncula* and primmorphs formed from this species. (**A**) Specimens maintained in aquaria; (**B**) Single cells were prepared and (**C**) allowed to reaggregate to 3D-cell aggregates, the primmorphs, for three days in the incubation medium.

**Figure 2:**
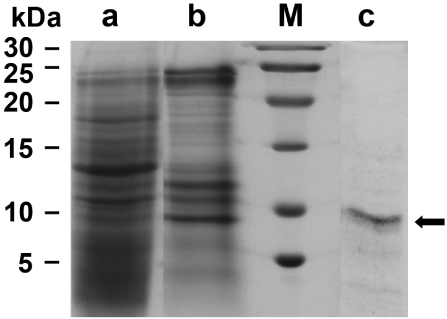
Analysis of the fractions, containing Suberitine, obtained during the purification procedure by SDS-PAGE (15% acrylamide/0.1% SDS); 3 µg of protein were applied per slot. (**lane a**) Fraction II obtained after ammonium sulfate fractionation; (**lane b**) Fraction III after gel chromatography on Sephadex G-100; (**lane c**) Fraction IV after a final gel filtration chromatography step (Sephadex G-50). The arrow marks the single protein band of an apparent size of 7000 to 8000: (**M**) Size markers.

Suberitine was purified from freshly collected animals (Figure 1A). Using a purification procedure, basically proposed by [19], a 55.8-fold enrichment was obtained using the consecutively performed steps of ammonium sulfate precipitation, Sephadex G-100 and Sephadex G-50 gel chromatography, as outlined under “Materials and Methods”. During this procedure the specific hemagglutination activity increased from 469.6 units/mg to 26,219.1 units/mg (Table 1). The purity of the fractions was analyzed by SDS-PAGE. Likewise the samples were subjected to SDS treatment and heat. It was seen that both Fraction II (ammonium sulfate fractionation; Figure 2, lane a) and Fraction III (Sephadex G-100 filtration) contained many protein bands (Figure 2, lane b), while after fractionation by Sephadex G-50 the pooled fractions that contained the majority of the hemagglutination activity displayed only one band corresponding to an apparent size of 7000 to 8000 Da (Figure 2, lane c). This fraction (0.29 mg protein/mL) was used for the following experiments.

**Table 1:** Purification of Suberitine. The crude extract started from 50 g fresh tissue.

Fraction	Total Protein (mg)	Total Hemagglutination (units)	Purification (fold)	Recovery (%)	Specific Activity (units/mg)
**I:** Crude fraction	218.4	102,370	1	100	469.6
**II:** (NH_4_)_2_SO_4_ precipitation	13.02	47,940	7.8	47	3687.7
**III:** Sephadex G-100	3.24	32,640	21.4	32	10,074.1
**IV:** Sephadex G-50	1.05	27,530	55.8	27	26,219.1

### 2.2. Suberitine: Biological Activity

As reported, the purified Suberitine displayed a pronounced biological hemagglutination activity [19,21]. 

**Figure 3:**
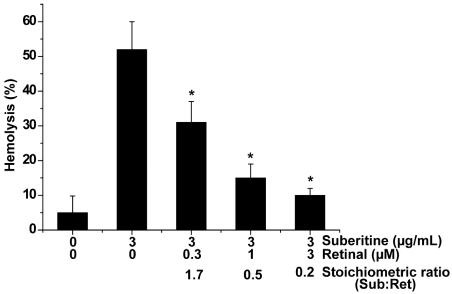
Abolishment of the hemagglutination activity of Suberitine by retinal. Constant concentrations of Suberitine (3 µg/mL, equal to 0.4 µM of the purified peptide) were incubated with 0.3 to 3 µM of retinal and then added to the hemolytic test system. For the evaluation of the hemagglutination activity an assay system was used, allowing a percental assessment of the degree of hemolysis, as described under “Materials and Methods”. The values represent the means ± SEM of ten experiments each; *P < 0.05.

In order to determine the interacting effect of metabolites of the β-carotene/retinoic acid pathway in sponges, a modified hemagglutination assay was applied, allowing an assessment on a percental basis. Among the metabolites tested (β-carotene, *all-E*-retinal, *all-E*-retinol and *all-E*-retinoic acid) *all-E*-retinal was the strongest interactor molecule, on the stoichiometric basis, reducing the hemagglutination activity of Suberitine at a [molar] stoichiometric basis (Suberitine/retinoid). At a molar ratio of 1.7 (Suberitine/retinal) the activity was lowered significantly by 40%, from 52 ± 8% down to 31 ± 6% (Figure 3); in comparison the reduction by β-carotene was 27%, by *all-E*-retinol 34% and by *all-E*-retinoic acid 19%, under the same concentration ratio conditions. Increasing the concentration of retinal and leaving the level of Suberitine constant in the assay, and by that decreasing the molar ratio (Suberitine/retinal) to 0.5, resulted in an about 40% reduction of the hemagglutination activity, down to 15 ± 4%. A further increase of retinal (ratio of 0.2) augmented the reducing activity of retinal to 10 ± 2%. In the absence of Suberitine or the presence of 3 µM retinoid, no significant hemolysis was seen under the conditions used (Figure 3 and not shown here).

These data suggest that retinal interferes with Suberitine and reduces the hemagglutination activity of this peptide. 

### 2.3. Interaction of Suberitine with Retinal

**Figure 4:**
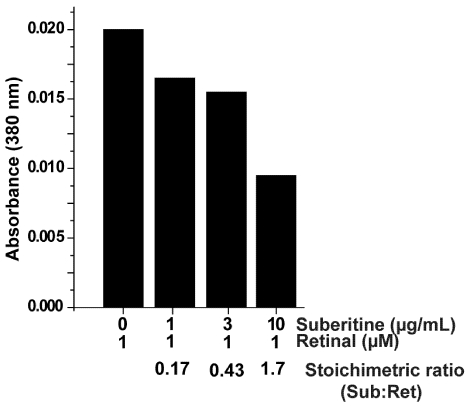
Effect of Suberitine on the absorbance spectrum of retinal, here measured at the wavelength of 380 nm.

In order to assess the (potential) interfering effect of retinal with Suberitine, the optical absorption spectrum at 380 nm was followed after addition of the components. For the assay, the concentration of retinal remained unchanged, while the concentration of Suberitine was increased (Figure 4). Under the conditions used, 1 µM of retinal showed an absorbance of 0.02, a value that agrees with the extinction coefficient of 42,800 M^−1^ cm^−1^ that had been measured in ethanol [26]. Addition of Suberitine at a molar ratio (Suberitine:retinal) of 0.17 caused a reduction to 0.016; a further increase of the concentration of Suberitine in the assay caused a further reduction of the absorbance value of retinal, down to 0.01 at a 1.7 molar ratio. Suberitine alone showed no measurable absorbance at 380 nm. 

### 2.4. Effect of Suberitine on the Viability of Sponge Cells

**Figure 5:**
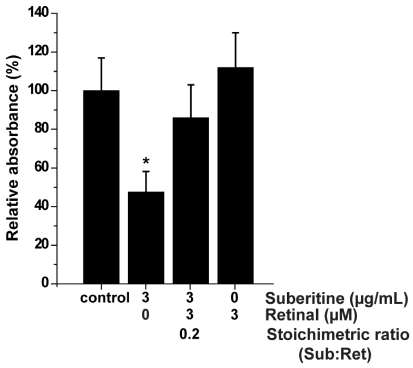
MTT assay was used to determine viability of *S. domuncula* cells treated with Suberitine, in the absence or presence of retinal. Each data point represents the means ± SEM (*n* = 10); *P < 0.05 correlated to the controls.

The effect of Suberitine on the viability of sponge cells was determined after an incubation period of 24 h by applying the MTT assay system. The amount of formazan formation was used as an indicator for metabolically active mitochondria in viable cells. The cell viability in assays not subjected to either Suberitine or retinal was set to 100% (Figure 5). Addition of 3 µg/mL of Suberitine caused already after the 24 h incubation period a significant reduction of cell viability by 46%. This inhibitory effect was abolished by retinal at a stoichiometric ratio of 0.2 (Suberitine/retinal). Retinal alone displayed no effect on cell viability.

### 2.5. Molecular Cloning of the Two (Related) Carotenoid Oxygenases

**Figure 6:**
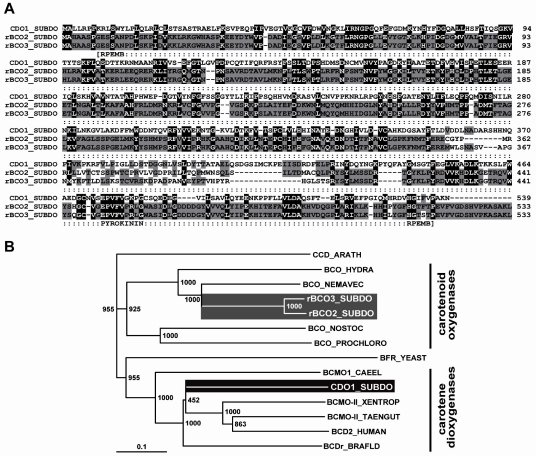
*S. domuncula* related β-carotenoid oxygenases. (**A**) The two sponge putative carotenoid oxygenases (rBCO1_SUBDO; rBCO2_SUBDO) were aligned with the *S. domuncula* carotene dioxygenase (CDO1_SUBDO). Residues conserved (identical or similar with respect to their physico-chemical properties) in all three sequences are shown in white on black; those that share similarity only in two sequences are in black on gray. The characteristic signatures, the pyrokinin signature [pyrokinin] and the signature for the retinal pigment epithelial membrane protein superfamily [RPEMB], are marked; (**B**) For phylogenetic analyses, the three sponge oxygenases were aligned with the two cnidarian putative β-carotenoid oxygenases from *N. vectensis* (BCO_NEMAVEC) and from *H. magnipapillata* (BCO_HYDRA) together with the carotenoid oxygenase from the cyanobacteria, from *N. punctiforme* (BCO_NOSTOC) and from *P. marinus* (BCO_PROCHLORO). Additionally, selected carotene dioxygenases have been included; from human (BCD2_HUMAN; EAW67189.1), from *Xenopus tropicalis* (BCMO-II_XENTROP; XP_002942801.1), from *Taeniopygia guttata* (BCMO-II_TAENGUT; XP_002189781.1), from *Branchiostoma floridae* (BCDr_BRAFLD; XP_002605888.1) and from *Caenorhabditis* *elegans* (BCMO1_CAEEL; NP_496729.2), as well as the yeast Bfr2p from *Saccharomyces cerevisiae* (BFR_YEAST; EGA87437.1). As an outgroup sequence to root the tree, the 9-cis-epoxycarotenoid dioxygenase from *Arabidopsis thaliana* (CCD_ARATH; NP_191911.1) has been selected. The degree of support of internal branches was assessed by bootstrapping (1000 replicates) and the evolutionary distance was calculated (0.1 aa substitutions per position in the sequence).

The complete cDNAs (*SDrBCO1* and *SDrBCO2*), encoding the *S. domuncula* related β-carotenoid oxygenases, termed rBCO1_SUBDO and rBCO2_SUBDO, were isolated. The sizes of the nt sequences were 1763 bp for *SDrBCO1* and 1772 bp for *SDrBCO2*. The completeness of the cDNAs was proven by Northern blot analysis (1.8 kb; data not shown). The two nt sequences comprise one ORF each, coding for putative 533 aa long polypeptides with a size of 60,519 Da and a pI [isoelectric point] of 8.53 for rBCO1_SUBDO; for rBCO2_SUBDO the calculated values are 60,258 Da and a pI of 8.60. The two new polypeptide sequences share a similarity/identity of 90%/89% to each other and 21%/37% between rBCO1_SUBDO and the recently identified sponge CDO1_SUBDO sequence (accession number FR848951) [20%/36% rBCO2_SUBDO to CDO1_SUBDO]. The polypeptides comprise the following signatures: the pyrokinins signature (aa_449_ to aa_457_ [with respect to rBCO1_SUBDO]), characteristic for insect neuropeptides; and the signature for the retinal pigment epithelial membrane protein superfamily (aa_8_ to aa_517_; expect value = 2.4e^−29^). Both motifs are also found in the sponge CDO1_SUBDO sequence (Figure 6A). In the following experiments, the sequence of the related carotenoid oxygenase rBCO2_SUBDO (*SDrBCO2*) was selected together with the carotene dioxygenase (CDO1_SUBDO/*SDCDO1*). (Database: The following sequences have been deposited in the EMBL/GenBank database; the *Suberites domuncula* mRNA for the putative β-carotenoid oxygenase (BCO1 gene) with the accession number HE603240, and the *S. domuncula* putative β-carotenoid oxygenase (BCO2 gene) with HE603239.)

The highest sequence similarity of the sponge related carotenoid oxygenase was identified with the two cnidarian putative β-carotenoid oxygenases from *Nematostella vectensis* (sea anemone; XP_001635106.1) and from *Hydra magnipapillata* (fresh water polyp; XP_002163128.1) with E-values of <2e^−125^. No similar sequence could be identified among more evolved metazoan taxa, having an E-value of <e^−20^. However, significant similarity was found by searching the databases to carotenoid oxygenase from cyanobacteria, e.g., from *Nostoc punctiforme* (YP_001864026.1; E-value = 4e^−96^), or from *Prochlorococcus marinus* (NP_874706.1; E-value = 4e^−53^). A phylogenetic tree was constructed with the two sponge and the two cnidarian carotenoid oxygenases together with the sponge carotene dioxygenase and some selected related polypeptides. After alignment and application of the distantly related 9-*cis*-epoxycarotenoid dioxygenase from *Arabidopsis thaliana* (NP_191911.1) as an outgroup the rooted tree showed that the β-carotenoid oxygenases and the carotene dioxygenases fall into two branches (Figure 6B). Among the carotenoid oxygenases the eukaryotic oxygenases are separated from the prokaryotic oxygenases. 

### 2.6. Determination of Oxygenase Activity in Bacterial System

**Figure 7:**
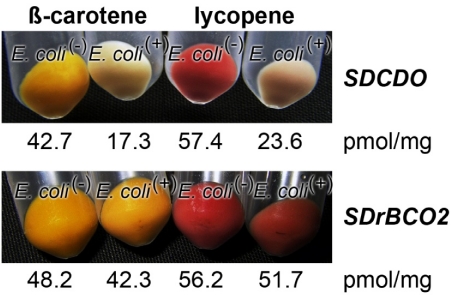
Proof that the newly described oxygenase gene *SDrBCO* does encode an enzyme that can metabolize neither β-carotene nor the related carotenoid, lycopene, by application of the color shift *E. coli* system. In a positive control series, (**Upper Panel**) the β-carotene synthesizing *E. coli*^(*β-car+*)^ or lycopene producing *E. coli*^(*lyco+*)^ strains were additionally transformed with the sponge *SDCDO* gene (*E. coli*^(*+*)^), while the controls remained empty (*E. coli*^(*−*)^). After induction with L-(+)-arabinose the super- transformed strain (*E. coli*^(*β-car+*)^ [*E. coli*^(*lyco+*)^]:*E. coli*^(*+*)^ system) remained whitish, due to the metabolic cleavage of either β-carotene or lycopene, while the empty strain (*E. coli*^(*β-car+*)^ [*E. coli*^(*lyco+*)^]:*E. coli*^(*−*)^ system) became colored yellowish or reddish and contained high amounts of the respective retinoid β-carotene or lycopene. Testing this system (*E. coli*^(*β-car+*)^ or lycopene producing *E. coli*^(*lyco+*)^) with the new oxygenase gene *SDrBCO* (**Lower Panel**) both the super-transformed strains, carrying the *SDrBCO* gene (*E. coli*^(*β-car+*)^ [*E. coli*^(*lyco+*)^]:*E. coli*^(*+*)^ system) or lacking it (*E. coli*^(*β-car+*)^ [*E. coli*^(*lyco+*)^]:*E. coli*^(*−*)^ system) have the same color and contain almost the same amount of retinoid, either β-carotene or lycopene, respectively.

The function of the *S. domuncula* *SDrBCO* was examined in *E. coli*, which had been transformed with the gene clusters maintaining the accumulation of either β-carotene [*E. coli*^(*β-car+*)^] or lycopene [*E. coli*^(*lyco+*)^] in the microorganisms. These strains were subsequently additionally transformed with the *S. domuncula* *SDrBCO* gene inserted into *pBAD-TOPO*. As a positive control, the carotinoid synthesizing bacteria were transformed with *SDCDO* [16]. The bacteria, lacking the sponge oxygenase genes, either *SDrBCO* or *SDCDO*, termed *E. coli*^(−)^, turned to a yellowish (for *E. coli*^(*β-car+*)^) or reddish color (for *E. coli*^(*lyco+*)^) due to the accumulation of β-carotene or lycopene, respectively (Figure 7). The *E. coli* strain carrying the *S. domuncula SDCDO* gene remained after incubation almost white or gray, indicating that those microorganisms metabolized the blue color absorbing retinoid metabolites β-carotene or lycopene, respectively. This visual assessment was supported by a quantitative analysis of the amount of β-carotene (or lycopene) in the respective cultures. A quantitative analysis of the amount of β-carotene present in overnight cultures of *E. coli*^(*−*)^, which did not carry the *SDCDO* gene, revealed a concentration of 42.7 pmol/mg (dry matter), while the bacteria that had been transformed with the *SDCDO* gene contained only 17.3 pmol/mg of β-carotene. The same effect is seen for the *E. coli*^(*lyco+*)^ system; the *SDCDO* gene-lacking bacteria contained 57.4 pmol/mg lycopene, while the double-transformed bacteria had 23.6 pmol/mg (Figure 7). 

In contrast, the transformed *E. coli* strains (*E. coli*^(*β-car+*)^ or *E. coli*^(*lyco+*)^) that had been super-transfected with the new carotenoid oxygenase gene, *SDrBCO2*, showed both the same coloration and almost the same amount of β-carotene (*E. coli*^(*β-car+*)^:*E. coli*^(*+*)^ system) or lycopene (*E. coli*^(*lyco+*)^:*E. coli*^(*+*)^ system), like the microorganisms not super-transfected with *SDrBCO2* (*E. coli*^(*−*)^). Based on these findings we conclude that the carotenoid oxygenase 2 gene (*SDrBCO2*) does not encode an enzyme that is able to metabolize either β-carotene or lycopene (Figure 7). Therefore, we termed these new oxygenases “related carotenoid oxygenases”.

### 2.7. Effect of Light on the Expression of the Carotene Dioxygenase and the Carotenoid Oxygenase 2

The steady-state expression levels of the two *S. domuncula* oxygenase genes, *SDCDO1* and *SDrBCO2* has been determined in primmorphs, a special form of cell aggregates (Figure 1C) that developed during reaggregation from single cells (Figure 1B), after exposure to light of different wavelengths. The primmorphs were incubated either in the absence or presence of 3 µM β-carotene. One day prior to the experiments the primmorphs were kept in complete darkness. Three days after starting the cultivation of the aggregates they were exposed to light of different wavelength spectra for blue, green and red light. After an exposure period of 3 days the expression levels were determined by the technique of qPCR and correlated to the expression of the house-keeping gene *ß-tubulin*. 

**Figure 8:**
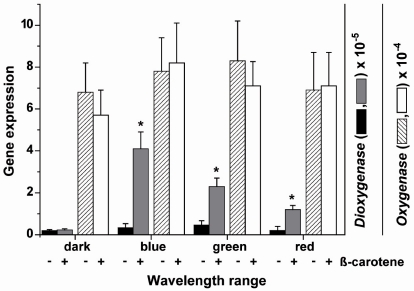
Expression of the *carotene dioxygenase* and the *carotenoid oxygenase 2* gene in primmorphs. The aggregates remained in the absence of or were incubated with 3 µM β-carotene. Three days after formation of the primmorphs the aggregates were exposed to blue, green or red light for an additional 3 days. Then the steady-state level of *carotene dioxygenase* and of *carotenoid oxygenase 2* was determined by qPCR using *tubulin* as reference gene. The expression levels are shown of the two oxygenases of primmorphs kept either in the absence (−) or presence (+) of 3 µM β-carotene and exposed to light of different wavelengths. The increase of the *carotene dioxygenase* expression is significant (*P < 0.05 referred) in primmorphs if the light exposed cultures had been incubated also with β-carotene. The bars represent the means (±SD) from 10 separate experiments each.

In the dark the expression level of the *carotene dioxygenase* is low with approximately 0.2 × 10^−5^ transcripts with respect to that of the house-keeping gene *tubulin*. Addition of β-carotene has no effect on the expression level of the *dioxygenase* in the dark. Exposure of the primmorphs not incubated in the presence of β-carotene, to light of different wavelengths showed a non-significant increase in the level of transcripts. However, if the β-carotene-treated primmorphs were exposed to light a significant increase of the expression level was seen. The level of the *dioxygenase* expression increased by 10.5-fold if exposed to blue light, by 5.1-fold with green light and by 4.9-fold after exposure to red light (Figure 8). 

The expression level of the *carotenoid oxygenase 2* in primmorphs in the dark was higher (around 7 × 10^−4^, correlated to the level of *tubulin*), compared to that of the *dioxygenase* (Figure 8). This level did not change if the primmorphs were incubated with β-carotene or not (Figure 8; only the values for the expression in the dark are shown). However, in contrast to the *carotene dioxygenase*, the steady-state expression of the *carotenoid oxygenases 2* in the primmorphs did not change significantly, if the aggregates were exposed to blue, green or red light (Figure 8), irrespective if they were β-carotene treated or not (data not shown).

## 3. Discussion

Suberitine is a neurotoxic (for crabs) and hemolytic (for mammalian erythrocytes) protein [22] which is even cytotoxic for the cells of the producer animal, *S. domuncula*, as shown in this study. Therefore, this sponge had to develop a mechanism that controls the potential self-directed bioactivity within the own body. Until now the genetic blueprint for Suberitine is not known. Therefore, it was not yet possible to elucidate if Suberitine is activated after translation, perhaps by formation of disulfide linkage or by proteolytic processing of a biologically inactive pro-toxin. 

**Figure 9:**
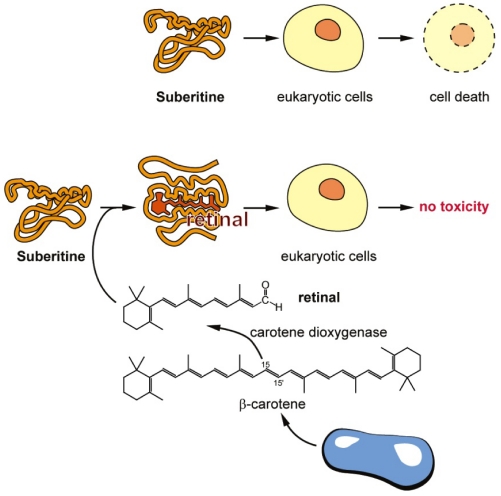
Proposed interaction of retinal with Suberitine (scheme). (**Upper panel**) In the absence of retinal Suberitine interacts with eukaryotic cells by killing them either by hemolysis or by (likely) necrotic cell death; (**Lower panel**) Retinal interacts with Suberitine by non-covalent, reversible energy transfer reaction under formation of a complex that is not toxic for eukaryotic cells; (**Bottom**) The route of formation of retinal from β-carotene that is produced in bacteria is outlined.

Due to the preliminary work by Cariello and Zanetti [24], describing a *S. domuncula* blue carotenoprotein, it was suggestive to postulate a connection between retinoids and Suberitine, an association that might modulate the bioactivity. In a recent study it had been shown that *S. domuncula* cells and tissue are especially rich in β-carotene and retinal [16]. While β-carotene is a product of the sponge-associated microorganisms, retinal is the enzymatically cleaved metabolite formed by the host-encoded carotene dioxygenase. The concentration of β-carotene in freshly collected specimens from the field is high with 137 ± 12 nmol/g dry tissue (carrots in comparison contain about 300 nmol/g [27]), and drops in animals kept in aquaria to 11 ± 4 nmol/g in parallel with the decrease of the load with bacteria [16]. In view of the findings that (i) the tryptophyl residues within the Suberitine molecule are crucial for its activity [23] and (ii) that retinal binds to tryptophan residues in some proteins, e.g., rhodopsin [28], by a reversible energy transfer reaction, we studied the quenching effect of Suberitine on retinal on a molar stoichiometric basis. The data revealed that a distinct reduction of the absorbance of retinal occurred at 380 nm at a stoichiometric Suberitine/retinal ratio of ≥1, suggesting a non-covalent interaction between these two components. This assumption is supported by gel chromatographic studies, which revealed that Suberitine, incubated with retinal and subsequently chromatographed with a 1 M NaCl containing PBS buffer through the Sephadex G-50 matrix, had the same elution behavior like untreated Suberitine (data not shown). This spectroscopic finding was confirmed by functional studies as well. Both the hemolytic activity and the cell toxicity of Suberitine were significantly reduced by retinal at a stoichiometric Suberitine/retinal ratio of around 1. The interplay of retinal with especially light-sensitive retinylidene proteins is well studied [29]. This interaction can be attributed to the retinal-mediated protein conformation changes caused by tryptophan-retinal coupling and subsequent quenching [30]. From these *in vitro* data we conclude that retinal controls the activity of Suberitine at a stoichiometric ratio of around 1. Moreover, we propose that also *in vivo* retinal modulates the biological activity of Suberitine. This view would imply that the β-carotene level, and in turn also the retinal level, affects the self-directed toxicity of the toxin (Figure 9). If the level of the retinoid is high the toxin displays no cytotoxic effect on the host producer cells, while at lower levels of these metabolites Suberitine also causes self-directed cell toxicity. This proposed mechanism may not be as far-fetched as it sounds at first glance. From previous studies it is well established that in *S. domuncula* self-destruction of tissue occurs after (i) tissue impairment in response to low aeration or after the adverse influence of xenobiotics [10] or (ii) as allograft reaction [31]. These two processes are the results of (self-directed) apoptotic responses. The self-directed cell toxicity caused by Suberitine is likely caused by necrosis, since no apoptotic DNA fragmentation could be indentified in sponge cells, exposed to the homologous toxin.

From previous studies it is known that the retinoids (existing in sponges) in the species *S. domuncula* are derived from β-carotene through sponge-associated/symbiotic microorganisms [15,16]. In more evolved metazoans, e.g., in vertebrates, this pro-vitamin A carotenoid is either symmetrically cleaved (via the β,β-carotene-15,15′-dioxygenase) to retinal [25] or asymmetrically metabolized (via the asymmetrically cleaving by β-carotene dioxygenases) to β-apo-10′-carotenal and β-ionone [32]. Since in sponges, until now only the β,β-carotene-15,15′-dioxygenase has been identified and cloned [16], a screening for potential additional genes that might be involved in the β-carotene metabolism was needed. Searching the *S. domuncula* database that comprises over 40,000 sequences [33], two clusters of ESTs (Expressed Sequence Tag) sequences were found whose deduced proteins were closely related to carotenoid oxygenases. The cDNA sequences of both groups were completed. A phylogenetic analysis of the resulting two deduced polypeptides revealed that these new sponge polypeptides share highest sequence similarity to the hydrozoan hypothetical oxygenases from *N. vectensis* and from *H. magnipapillata*. Surprisingly, no further related polypeptides could be identified in higher metazoans; however, distinct similarities exist to cyanobacterial carotenoid oxygenases. Since the two new sponge oxygenases are highly similar in their sequences (90% on the protein level), only one of them, the rBCO3_SUBDO (*SDrBCO3*) was selected for the further analyses. The fact that these mentioned sponge genes/cDNAs, encoding for proteins involved in retinoid metabolism, have been (initially) identified in an EST (Expressed Sequence Tag) database from *S. domuncula* (see [10]) implies that the cDNAs were obtained from poly(A)^+^ mRNAs, sequences that exist only in multicellular eukaryotic organisms. Hence, already this piece of evidence supports that these genes are described from sponges derived from the *S. domuncula* host and not from associated microorganism. Furthermore, the phylogenetic analyses of the deduced proteins, given here and previously [16], clearly identify the sequences as sponge-derived. 

In order to verify a potential β-carotene-degrading tomato carotenoid, lycopene [34], metabolizing activity that might be caused by the new sponge oxygenase, bacterial β-carotene- and lycopene-synthesizing and accumulating *E. coli* strains were used [32,35]. The data revealed that *E. coli* that had been transformed with the new sponge oxygenase did not degrade β-carotene or lycopene. In turn, the new sponge oxygenases were termed “related carotenoid oxygenases”. Based on these findings we suggest that the new related carotenoid oxygenase from *S. domuncula* might hydrolyze other retinoids in sponges. From studies in this animal it is known that a series of retinoids, retinal, retinol or retinoic acid, exists that is distinctly different from β-carotene [15,16]. Furthermore, the high abundance of carotenoids in sponges is well described [36]. 

Published studies described that in *S. domuncula* retinoic acid acts as a morphogen during the development of aquiferous canals [17] and as an inducer of the bone morphogenetic protein-1, required for the processing of silintaphin-2 during spicule formation [16]. Since *S. domuncula* comprises a light-reacting protein network with the luciferase as light-producing [37] and the cryptochrome as a (potential) photon-responding molecule [38], our group intensively screened for light sensory photopigments. β-Carotene, retinol and retinal are surely candidates that might act as putative cofactor(s) of the suspected photopigment protein. Therefore, we incubated primmorphs with β-carotene, retinol or retinal, and determined their effect on the expression of the carotene dioxygenase as well as on the new described, related carotenoid oxygenase in dependence on light of different wavelengths. From such a study we wanted to obtain a first indication if the enzymatic processing pathway of β-carotene, either β-carotene itself or the retinal/retinol metabolites, is linked to distinct light wavelengths. The data revealed that indeed light causes a differential effect on gene expression of the dioxygenases. At first, it was established that only the expression of the *carotene dioxygenase* is influenced by light, while the steady-state level of the *carotenoid oxygenase* gene remained unchanged. This effect is seen only in those primmorphs that had been incubated with β-carotene. Primmorphs that were treated with retinol or retinal showed a much lower response to light (data not shown). In the dark the level of the *carotene dioxygenase* transcripts was relatively low. Exposure to light significantly increased the expression of the *carotene dioxygenase*; especially the response to blue light is high (10.5-fold) compared to the expression in the dark. Red or green light was less effective. This finding can be explained on the basis of the habitat where *S. domuncula* lives. In the sea these animals are found in a depth of about 20 m, an environment that is predominantly penetrated by the shorter wavelength spectrum of visible light, of around 380 to 500 nm (blue) [39]. Hence, the *carotene dioxygenase* gene is, after the *cryptochrome* [38], the second sponge gene whose expression is light-controlled. To the best of our knowledge no report has been published showing that the expression of the *carotene dioxygenase* in metazoans is light sensitive. In contrast, in plants the gene encoding the related 9,10/9′,10′ carotenoid cleavage dioxygenase is light-inducible [40]. Since the blue light-dependent increase of the sponge gene expression is only seen after incubation of the primmorphs with β-carotene we take this finding as a first evidence that β-carotene, or a deriving metabolite of it, is implicated in the light-reacting protein network in sponges.

## 4. Experimental Section

### 4.1. Sponge and Primmorphs

Specimens of *S. domuncula* (Porifera, Demospongiae, Hadromerida) were collected in the Northern Adriatic Sea near Rovinj (Croatia) at depths between 18 and 25 m, and then kept in aquaria in artificial seawater (Tropic Marine, Tropic Marine Centre Ltd., Rickmansworth; UK) in Mainz (Germany) at 17 °C [41]. For the experiments animals that had been kept in the aquarium for over three months were used. From those specimens primmorphs, 3D cell aggregates were prepared as described [16,42] and cultivated in natural seawater (Sigma-Aldrich, Taufkirchen; Germany) supplemented with 1% RPMI 1640 medium (Sigma). Where indicated, the primmorphs were incubated with 1 µM β-carotene (Sigma; C4582).

### 4.2. Exposure of Primmorphs to Light

The experimental design was given previously [43]. In short, primmorphs were prepared and kept for one day in complete darkness; at day 3 the aggregates were used for the experiments. Then the primmorphs were exposed to light of different wavelength ranges from a distance of 5–6 cm by using optical filters (Schott-Advanced Optics, Mainz, Germany) that were placed in front of a white light bulb [50 m^2^ kg/s^3^ (General Electric, Fairfield, CT, USA) 29208 projector white light bulb]. The following filters were used: for blue light a DMZ 12 filter (cutoff below 400 nm; maximum at 490 nm; cutoff above 599 nm), for green light VG9 (400 nm maximum at 520–650 nm), and red light KMZ 50 OG590 (700 nm maximum at 780–900 nm). The controls remained in the dark. Where indicated, the primmorphs were incubated from the beginning of the re-aggregation from single cells with 3 µM β-carotene.

### 4.3. Purification of Suberitine

For the isolation and purification of Suberitine the procedure of Cariello and Zanetti [19,24] has been used with modifications adopted from Müller *et al.* [44]. Sponge cubes were extracted in Ca^2+^- and Mg^2+^-free seawater; the suspension was centrifuged (5 °C; 80,000 × *g*; 30 min). Starting from 50 g of tissue a fraction (Fraction I) of 78 mL (2.8 mg protein/mL) was obtained. This fraction was subjected to ammonium sulfate precipitation (final concentration 80%). The precipitate formed was collected by centrifugation (5 °C; 80,000 × *g*; 30 min) and dissolved in 3 mL of PBS (pH = 7.4; 137 mM NaCl, 10 mM Na-phosphate, 2.7 mM KCl). The resulting Fraction II (3.1 mL; 4.2 mg protein/mL) was dialyzed exhaustively against PBS. Subsequently Fraction II was fractionated (in aliquots of 1 mL) by gel chromatography on Sephadex G-100 (Sigma-Aldrich; column size: diameter 1 cm, height 40 cm; fraction volume 1.2 mL). The fractions containing the hemagglutination activity, which eluted at a ratio V_e_/V_o_ (elution volume/void volume) [45] of 2.2 to 2.4 (3.6 mL), were collected (Fraction III; 0.9 mg protein/mL) and finally purified by gel filtration using Sephadex G-50 superfine (Sigma-Aldrich; column size: diameter 1 cm, height 50 cm; fraction volume 1.2 mL), as described [19]. The fractions with the highest hemagglutination activity (3.6 mL) were determined at a V_e_/V_o_ ratio of 1.60 to 1.72 (Fraction IV; 0.29 mg protein/mL). 

### 4.4. SDS-PAGE Analysis

Sodium dodecyl sulfate polyacrylamide gel electrophoresis (SDS-PAGE) was performed as described [16]. Samples of 3 μg of protein were mixed with loading buffer (Roti-Load; Carl Roth, Karlsruhe; Germany), boiled for 8 min, and subjected to SDS-PAGE (15% acrylamide and 0.1% SDS). The gels were stained with Coomassie brilliant blue. The protein size standards (“Low Range Protein Ladder”) from Thermo Fisher Scientific (Rockford, IL USA) were used to estimate protein sizes.

### 4.5. Testing for Hemolytic Activity

The hemolytic action of Suberitine on red blood cells was tested with human type A erythrocytes as recently described [10]. In brief, a 10% (v/v) suspension of PBS-washed erythrocytes was exposed to serial dilutions of the respective Suberitine fraction in microtiter plates (Nunc/Thermo Electron, Langenselbold; Germany). For comparison and calibration of total hemolysis the samples were subjected to 1% (v/v) Triton X-100. After incubation (1 h, 37 °C) the plates were centrifuged (20 °C; 2000 × *g*; 10 min) and a 150 µL aliquot from the supernatant was taken and used for absorbance measurements at 450 nm. The reciprocal of the greatest dilution at which agglutination occurred was taken and given as titer (units) [46]. The values are related to the protein concentration in the sample.

For the competition experiments of Suberitine with retinal, we applied a different hemagglutination assay in order to assess the change of the activity on a percental basis [10]. The percentage of hemolysis was calculated as follows: (A_450_ of the Suberitine-treated sample − A_450_ of buffer-treated sample)/(A_450_ of Triton X-100-treated sample − A_450_ of buffer-treated sample) × 100%. 

In the co-incubation series of Suberitine with retinoids the purified peptide sample (either 3 µg/mL [0.4 µM]; or 1 µg/mL [0.14 µM]) was incubated with those compounds for 15 min at 37 °C and then used for the experiments.

### 4.6. Interaction of Suberitine with Retinal

In an earlier study it was clarified that besides cysteinyl residues, tryptophyl residues are also crucial for full activity of Suberitine [23]. Furthermore, it is known that retinal interacts with tryptophan residues in proteins [47], such as in the human retinol-binding protein, most likely by quenching the absorbance of the retinoid [48]. In order to assess the (potential) interfering effect of retinal with Suberitine the optical absorption spectrum at 380 nm was followed. 

### 4.7. Quenching of the Optical Absorption Spectrum of Retinal by Suberitine

The effect of retinal on the absorbance spectrum by Suberitine was measured optically using a Zeiss Spectrophotometer PMQ II at 380 nm, the maximum of the absorbance spectrum of retinal. The components were dissolved in PBS (pH 7.4). 

### 4.8. MTT Sponge Cell Viability Assay

The MTT (3-(4,5-dimethylthiazol-2-yl)-2,5-diphenyltetrazolium bromide) assay system, optimized for the determination of sponge cells, was applied [49]. Sponge single cells were obtained by dissociation in Ca^2+^- and Mg^2+^-free seawater; 200 µL each of a cell suspension (106 cells/mL, adjusted with a hemocytometer) were added to wells of microtiter plates and subsequently incubated for 24 h in natural seawater, supplemented with 1% RPMI 1640 medium. The incubation was terminated by addition of MTT (0.5 mg/mL) and the optical densities were measured at 495 nm (595 nm as reference wavelength) in an ELISA Reader (model 450; Bio-Rad). 

### 4.9. Molecular Cloning of *S. domuncula* (Related) Carotenoid Oxygenases

By searching the *S. domuncula* EST (Expressed Sequence Tag) database [33] several fragments were identified that encoded parts of a putative carotenoid oxygenase polypeptide. Their ORFs (open reading frame) were completed by the 3'- and 5'-racing technique using the CapFishing Full-length cDNA Premix Kit (Seegene; Rockville, MD, USA) as described [50]. Two different sequences were identified that were termed putative β-carotene oxygenases [BCO]-1 and -2. Since (as will be described later) these oxygenases do not cleave β-carotene, we termed these putative molecules “related to β-carotene oxygenases”. The complete nucleotide (nt) clones of the sponge related β-carotene oxygenases, *SDrBCO1* and *SDBrCO2*, comprise 1763 base pairs (bp) and 1772 bp, respectively, excluding the poly(A) streches; the ORFs rang from nt_45–47_ to nt_1644–1646(stop)_ and from nt_45–47_ to nt_1644–1646_. 

### 4.10. Sequence Analyses

The sponge sequences were analyzed with computer programs BLAST [51] and FASTA [52]. Multiple alignments were performed with CLUSTAL-W Ver. 1.6 [53] and the degree of support for internal branches was further assessed by bootstrapping [53]. Motif scan was performed using the internet database [54]. The graphic presentations were performed by using the Genedoc program [55].

### 4.11. Determination of the Potential Carotenoid Oxygenase Activity in the Bacterial System

Like in the recent study to prove that the sponge *SDCDO* cDNA encodes a carotene dioxygenase enzyme that cleaves β-carotene to retinal [16] by using the β-carotene- and lycopene-synthesizing and accumulating *E. coli* strains (*E. coli JM109* [32,35]), this bacterial system was also applied to clarify if the carotenoid oxygenases (*SDrBCO*), cloned here, metabolize this retinoid. These indicator *E. coli* strains were termed *E. coli*^(β-car+)^ and *E. coli*^(lyco+)^; the transformed strains become yellow/reddish since they form blue light absorbing β-carotene or lycopene, respectively. In the present study the bacteria were transformed with the *S. domuncula* gene *SDrBCO2* using the vector *pBAD-TOPO* (Invitrogen/Life Technologies, Darmstadt; Germany) following the described procedure [35]. Overnight cultures were induced with 0.5% (w/v) of l-(+)-arabinose for 3 h [32]. Subsequently the cells were collected, broken, and the amounts of β-carotene and lycopene were determined by reversed phase HPLC. In a parallel series of experiments the bacteria were transformed with the *SDCDO* cDNA [16] and used as a positive control. As one control, the strain lacking the genes for the oxygenase enzymes, named *E. coli*^(−)^, were used.

### 4.12. Quantitative Analysis of β-carotene and Lycopene

Defined amounts (wet weight) of broken bacterial cells were extracted with acetone. After evaporation identical aliquots (10 µL) were separated by reversed phase HPLC [35]. The quantification was achieved by calibration with peaks from defined amounts of β-carotene reference compound. 

### 4.13. Quantitative Real-Time RT-PCR (qRT-PCR)

The determination of the steady-state level of the *oxygenase* transcripts was performed by quantitative real-time RT-PCR (qRT-PCR) as described [16,44]. In brief, RNA was extracted and reverse transcribed and then subjected to qPCR in an iCycler (Bio-Rad; Hercules, CA). The following primer pairs were applied; for amplification of the *carotene dioxygenase* (accession number FR848951) Fwd: 5'-ATGCACGTTCTCATCACAATC-3' (nt_1088_ to nt_1108_) and Rev: 5'-GCACATAATACTCCCGTCACTC-3' (nt_1239_ to nt_1218_; size of the fragment, 152 bp) and for the amplification of the *S. domuncula* (*related) carotenoid oxygenase 2* (*SDrBCO2*) Fwd: 5'-TGGATACAAACTACCCTACAGAG-3' (nt_1__,__257_ to nt_1__,__279_) and Rev: 5'-ACATAGCCATCATCATCATCAC-3' (nt_1412_ to nt_1391_; 156 bp). The level of transcripts the house-keeping molecule *ß-tubulin* (AJ550806) was quantified with the primer pair Fwd: 5'-AACCGCTGTTTGCGACATCC-3' (nt_1126_ to nt_1145_) and Rev: 5'-CAATGCAAGAAAGCCTTTCGCC-3' (nt_1266_ to nt_1245_; 141 bp). The threshold position was set to 50.0 RFU above PCR subtracted baseline for all runs. Mean Ct values and efficiencies were calculated by the iCycler software. The estimated PCR efficiencies were in a range of 92%–103%. Expression levels of the respective transcripts for the *oxygenases* were correlated with the one for *ß-tubulin* to assess the relative expression levels (E_tubulin_^Ct oxygenase^/E_oxygenase_^Ct oxygenase^), whereby “E” describes the PCR efficiency and “Ct” represents the threshold cycle [56].

### 4.14. Additional Analytical Method

Protein concentrations were determined by the Bradford method [57] using Roti-Quant solution (Roth, Karlsruhe; Germany).

### 4.15. Statistical Analysis

The results were statistically evaluated using the paired Student’s *t*-test [58].

## 4. Conclusion

The data in the present study show that retinal controls the activity of the toxin Suberitine in its homologous system as well as in the vertebrate erythrocyte system. At a molar, stoichiometric ratio of 1:1 the retinoid abolishes the function of this toxin. The experiments suggest that the change of the biological activity is caused by a non-covalent interaction which, very likely, results in a change of the conformation of the protein. In turn, this is the first report which highlights that in sponges a low molecular bioactive metabolite can interact with a bioactive peptide and thus alters its function. This result also indicates that bioactive peptides/secondary metabolites in sponges might not only function as toxic agents but can also have the capacity to modulate the bioactivity of another toxic compound produced in the homologous system.
